# Which digital learning strategies do undergraduate dentistry students favor? A questionnaire survey at a German university

**DOI:** 10.3205/zma001631

**Published:** 2023-06-15

**Authors:** Anne Brigitte Kruse, Milena Isailov-Schöchlin, Marianne Giesler, Petra Ratka-Krüger

**Affiliations:** 1 University of Freiburg, Faculty of Medicine, Department of Operative Dentistry & Periodontology, Freiburg, Germany; 2 Freiburg, Germany

**Keywords:** digitization, distance learning, dental education, digital media

## Abstract

**Background:**

The development in teaching dental education toward ever greater digitization has gained enormous momentum in the last 2 years due to the pandemic. However, acceleration is not synonymous with improvement, especially from the learners' point of view. Therefore, the aim of this survey among students of dentistry was to determine which digital learning strategies and which media are preferred.

**Method:**

Undergraduate students of clinical semesters (6^th^ to 9^th^) in dental medicine during at the University of Freiburg participated in an online-survey. Questions were asked about personal learning strategies for and experience with using digital media for private and educational reasons. Furthermore, students were asked which digital learning formats they preferred for different learning phases.

**Results:**

Students (N=148) are experienced in using digital media for learning. They prefer classical media (such as textbooks and lectures) for acquiring basic theoretical knowledge and mention digital teaching formats more in relation to practical training and complex treatment procedures. 67% prefer learning alone and 90% rate visualizations as helpful for learning. 78% report, that they feel well supported in the learning process by digital media and 83% agree that e-learning offerings are a quality factor for university teaching. 82% state e.g. that the growing range of online content allows a more flexible approach to face to face-teaching, enriches classroom teaching (78%) and helps organize one’s own study (79%).

**Conclusion:**

Students have a positive attitude towards the use of digital media, especially when it comes to having more time available for practical exercises. They also see an advantage in the fact that through the use of digital media, lectures can be organized more flexibly and also the organization of their studies can be optimized. New digital teaching media should be created based on these results. It is important to consider which digital formats seem suitable for which content during different semesters.

## Zusammenfassung

**Hintergrund:**

Die Entwicklung in der zahnmedizinischen Ausbildung hin zu einer immer stärkeren Digitalisierung hat in den letzten zwei Jahren aufgrund der Pandemie eine enorme Dynamik erfahren. Beschleunigung ist jedoch nicht gleichbedeutend mit Verbesserung, insbesondere aus Sicht der Lernenden. Ziel dieser Umfrage unter Zahnmedizinstudierenden war es daher, zu ermitteln, welche digitalen Lernstrategien und Lehrmedien bevorzugt werden.

**Methode:**

Studierende des 6. bis 9. klinischen Semesters der Zahnmedizin an der Universität Freiburg nahmen an einer Online-Befragung teil. Es wurden Fragen zu persönlichen Lernstrategien und Erfahrungen mit der Nutzung digitaler Medien für private und ausbildungsbezogene Zwecke gestellt. Darüber hinaus wurden die Studierenden gefragt, welche digitalen Lernformate sie für verschiedene Lernphasen bevorzugen.

**Ergebnisse:**

Die Studierenden (N=148) waren beim Lernen erfahren im Umgang mit digitalen Medien. Sie bevorzugten klassische Medien (wie Lehrbücher und Vorlesungen) für den Erwerb von theoretischem Basiswissen und gaben digitale Lehrformate eher in Bezug auf die praktische Ausbildung und komplexe Behandlungsabläufe an. 67% bevorzugten selbstgesteuertes Lernen und 90% bewerteten Visualisierungen als hilfreich für das Verständnis. 78% gaben an, dass sie sich durch digitale Medien im Lernprozess gut unterstützt fühlen und 83% stimmten zu, dass E-Learning-Angebote ein Qualitätsfaktor für die Hochschullehre sind. 82% gaben z.B. an, dass das wachsende Angebot an Online-Inhalten eine flexiblere Gestaltung der Präsenzlehre ermöglicht, die Präsenzlehre bereichert (78%) und bei der Organisation des eigenen Studiums hilft (79%).

**Schlussfolgerung:**

Die Studierenden stehen dem Einsatz digitaler Medien positiv gegenüber, insbesondere wenn es darum geht, mehr Zeit für praktische Übungen zur Verfügung zu haben. Einen Vorteil sahen sie auch darin, dass durch den Einsatz digitaler Medien die Lehrveranstaltungen flexibler gestaltet und die Organisation des Studiums optimiert werden können. Auf der Grundlage dieser Ergebnisse sollten neue digitale Lehrmedien entwickelt werden. Dabei ist es wichtig zu berücksichtigen, welche digitalen Formate für welche Inhalte in den verschiedenen Semestern geeignet erscheinen.

## 1. Introduction

As a massive accelerator, the pandemic led universities worldwide to immediately adopt digital teaching formats of different kinds. Suddenly, for theoretical content, traditional classroom teaching seemed to be overcome and replaced by many more flexible formats for students and teachers. However, e-learning alone does not seem to be suitable for everyone and can negatively affect students' mental health [1]. A survey of 11,000 students and 1,800 teachers at German universities showed that satisfaction with the learning experience dropped from 85% during the winter semester 2019/20 (the beginning of the pandemic) to 51% during the following summer semester [2]. The reasons given here were a lack of social life among students, problems with motivation and concentration when studying at home, and inadequate opportunities for exchange with teachers [2]. Results of a meta-analysis on the mental health of dental students during the pandemic from countries across the world showed a prevalence of depression of 37% [3]. On the other hand, research conducted prior to the COVID pandemic found that blended-learning approaches such as flipped classroom were favored as a teaching method by dental students [4]. In particular, self-centered learning through online resources was cited by dental students as the greatest advantage of this teaching method. Therefore, the challenge of digitization is to use different digital learning formats and learning strategies that achieve a comparable learning effect and finds the right balance between synchronous and asynchronous learning [5]. Recent literature has focused on accelerated digitization and its advantages and disadvantages. Several authors have found that the basic acceptance of digital learning among students is high [6], [7], [8], [9]. Nevertheless, the use of digital technologies is not only associated with a solution for users, but also with a learning curve for students and also faculties, and students' anxiety about adapting to distance learning must also be taken into account [5]. At the same time, however, digital learning seems rather unsuitable, especially for practical teaching content in medical education [10], [11]. The need for further development of digital teaching appears to be essential, especially for practical content. The development of new digital teaching media requires evaluation, especially by the students, and subsequent improvement. Therefore, the opinion of the students as a target group on the didactically meaningful conception and use of e-learning elements in teaching plays a major role. Thus, didactically meaningful teaching scenarios that are also accepted by the students are needed. The Horizon Report, which annually reports on the framework conditions and trends of teaching and learning, also shows that for the year 2021, teleworking and tele learning spread in the social area, the differences between technically well-off and less well-off students were aggravated, and COVID-related restrictions negatively influenced the mental health of students [12]. On the technical side, the elements “artificial intelligence”, “learning analytics”, “blended & hybrid course formats”, “open educational resources” and “micro credentialing” are identified as particularly important.

To answer the question of effective learning strategies from the student's perspective, a survey of dental undergraduate students was conducted as part of this study. Second, this study determined which learning media were preferred by the students.

## 2. Materials and methods

### 2.1. Study design

An online-survey was made available via the common learning platform ILIAS, University of Freiburg, Germany. Invitations were sent to the students by email. The survey took place between December 2020 and October 2021.

### 2.2. Procedure

All undergraduate students of clinical semesters in dental medicine (6.-9. semester) at the University of Freiburg were asked to participate in a voluntary and anonymous survey. Students were asked to give their consent by ticking the required consent box. Consent was mandatory to start the survey. 

### 2.3. Questionnaire

We developed a questionnaire including questions about age, gender and number of semesters. Various questions were asked about personal learning strategies for and experience with using digital media for private and educational reasons. Furthermore, students were asked to assess different teaching strategies and which digital learning formats they preferred for different learning stages, such as basic knowledge, practical application, patient treatment, and advanced clinical treatment (e.g., complex case planning or implantology). Finally, there was a free text field for further suggestions for digital teaching formats or other aspects of dental education. For further details, see the full questionnaire in attachment 1 .

### 2.4. Statistical analysis 

For all variables we computed descriptive statistics. In order to determine whether there were differences between gender and semester groups with respect to learning and teaching strategy variables, preferences for learning media etc. we used χ^2^ tests, t-tests for independent samples or analyses of variance. Given the number of comparisons, Bonferroni adjustments were used.

Computations were performed using STATA (StataCorp LT, College Station, TX, USA, Version 17.0) and using the Statistical Package for Social Sciences (SPSS) version 26. 

## 3. Results

### 3.1. Demographic data

354 students from the clinical dentistry semesters were invited to participate in the survey, 155 responded. Due to incomplete questionnaire data, only 148 students were included in the analysis. The gender distribution of the included participants corresponds to the overall distribution of students of approximately 1/3 male and 2/3 female. For demographic data, see table 1 (Tab. 1).

### 3.2. General use of digital media

A total of 35.1% of all participants reported using e-learning monthly, and 55.4% indicated using e-learning occasionally e.g. before exams. Only 8.1% were using e-learning daily and 1.4% weekly. A total of 83.1% preferred using a personal computer or laptop; the next highest percentages were those of participants who preferred tablets (50.7%) and smartphones (10.8%). In response to the question of which teaching aids are best for learning, 55.4% mentioned textbooks and 73% lectures, while e-learning offers were ticked by 54.1% percent of the students.

For learning purposes most students have already gained experience with learning platforms (95.3%), cloud services for document exchange (93.9%), video conferences (89.9%), wikis (85.1%), communication services e.g. Skype, WhatsApp (77.7%), Google Docs (69.6%), research data basis (58.8%), digital services of the university library (45.3%) and social media (29.7%)

With regard to semester affiliation, students in the higher semesters were more likely to use databases for the enquiry of international articles than students in the lower semesters (χ^2^=29,194, p=.000). No other significant gender-specific differences were found for the use of media.

### 3.3. Digital media for private and learning purposes

The top ten digital media used for private reasons and in the context of learning are depicted in table 2 (Tab. 2), which shows that Facebook, Instagram and YouTube are the predominant digital media used for private purposes. Podcasts were moderately stated as a privately used medium. For learning purposes, students mainly use the learning platform ILIAS, which is offered by the university. A moderate proportion of students use the collaboration tool Google Docs and Wikipedia. Podcasts were favored by one fifth of the participants. The percentage of participants using forums is low. Contrary to their top positions among for private use, the positions of Facebook, Instagram and YouTube were at the bottom of the top ten digital media used for learning purposes.

### 3.4. Media types for specific learning purposes

**Figure 1:**
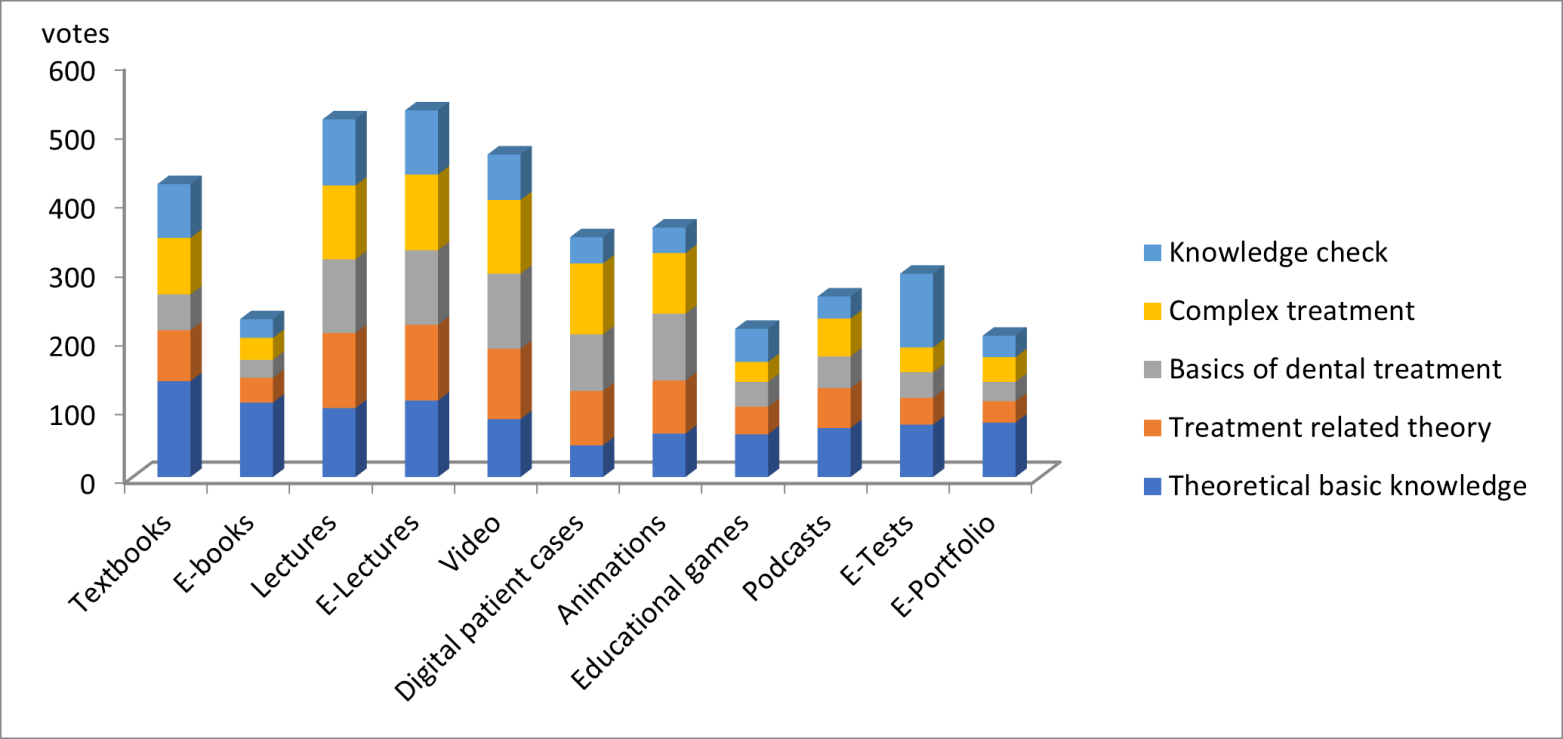
Media and learning purposes. This figure shows the cumulative number of votes for each category

The results for different media types with respect to specific learning purposes are shown in figure 1 (Fig. 1). Since multiple answers were possible for this question, a different total quantity for each category was obtained. Therefore, absolute numbers rather than percentages are described below. For the acquisition of theoretical knowledge, students preferred conventional textbooks (139), lectures (100). Additionally, a similarly high prevalence of e-books (108) and lecture recordings (111) appeared and a moderate proportion of videos (84), e-portfolios (79), e-tests (76) and podcasts (71) for acquiring theoretical knowledge could be found. For acquiring treatment-related theory there was a preference for digital media in addition to lectures (109). Recorded lectures (110) and videos (102) were also highly rated here. Similar results were obtained for teaching practical basics in dental treatment (108/109). For this purpose, animations and simulations were also favored (97). Concerning learning more advanced practical skills, digital patient cases show high popularity (103) next to lectures (107) and lecture recordings (110). Lectures (96) and lecture recordings (93) are also used to prepare for exams. Additionally, a high proportion favored electronic knowledge tests in this context (107).

### 3.5. Learning strategies

**Figure 2:**
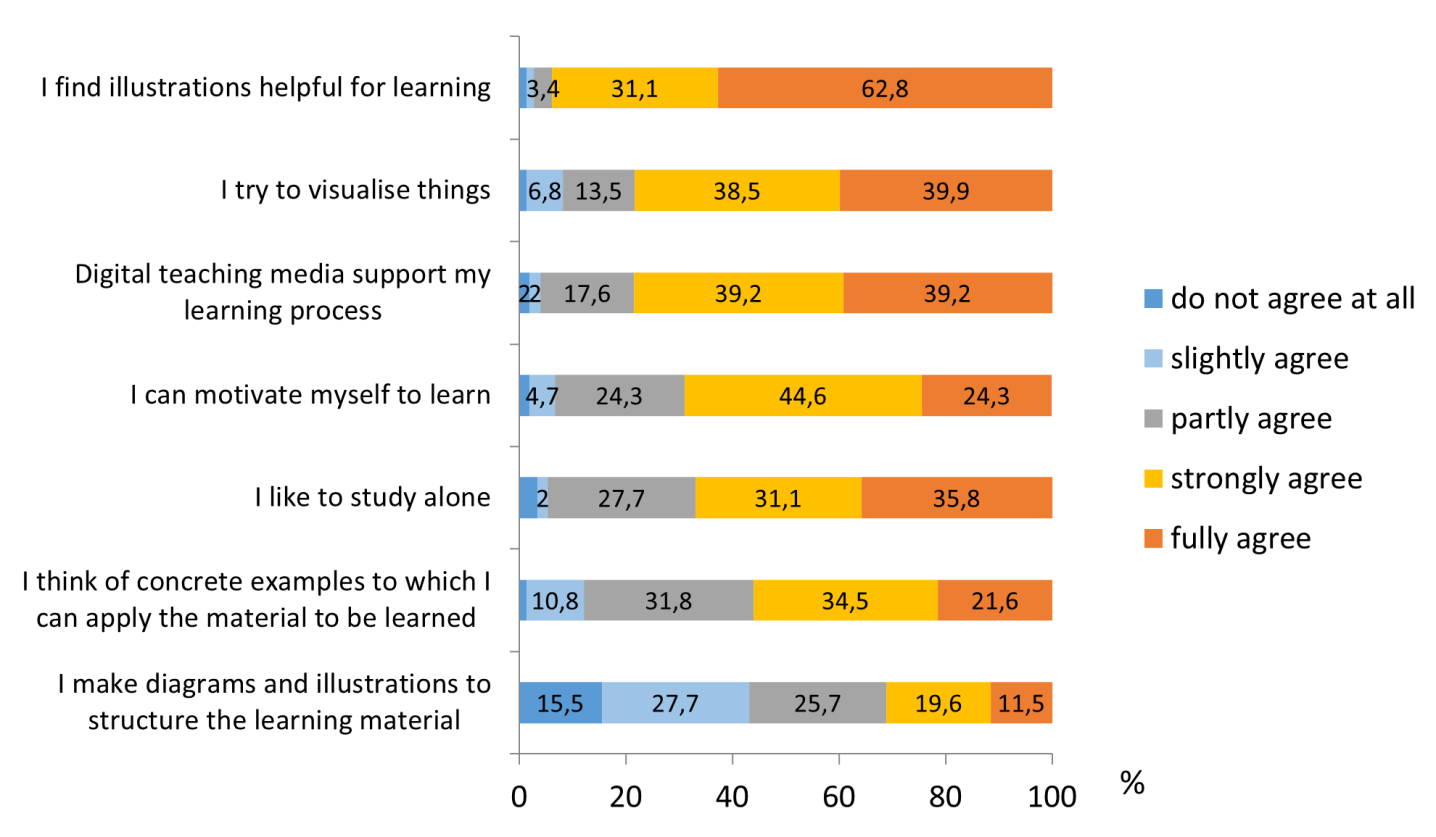
Extent to which selected learning strategies are used (%)

The self-assessment of one’s own learning behavior showed that a large percentage of participants tend to learn alone rather than in groups (66.9%). Respondents agree to a high degree that they can motivate themselves to learn (68.9%) and most of them report that they feel well supported in the learning process by digital media (78.4%). About 56 percent state that they think of concrete examples to which they can apply material to be learned. Furthermore about 94 percent report that the illustrations support them well in their learning process and about 78 percent confirmed using visualization to internalize learning content. On the other hand, only about 31 percent report that they make diagrams and illustrations to structure their learning material (see figure 2 (Fig. 2)).

### 3.6. Teaching strategies

**Figure 3:**
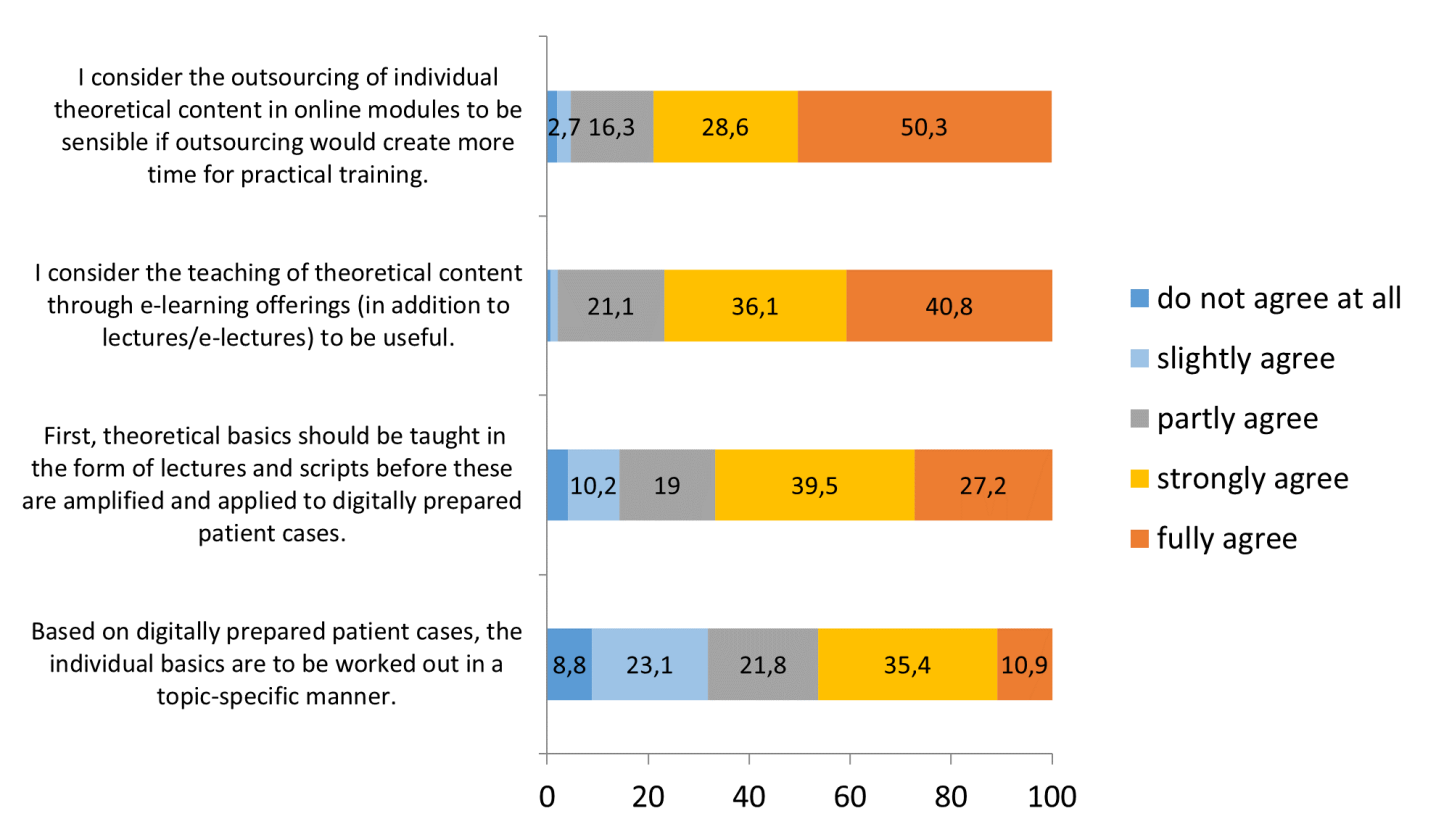
Assessments of teaching strategies (%)

With regard to the applied teaching strategy, about 67 percent of the students consider it more useful that first theoretical content should be taught in form of lectures and scripts before moving on to digitally prepared patient cases. Additionally, they find it most welcome when the teaching of theoretical content is expanded through e-learning offers (76.9%) and when the outsourcing of theoretical content to the digital area leads to more time for practical exercises (78.9%). Approximately 46 percent of the respondents believe it would make sense if the individual basics of digitally prepared patient cases are worked out in a topic-specific manner (see figure 3 (Fig. 3)).

### 3.7. Scope of e-learning now and in the future

**Figure 4:**
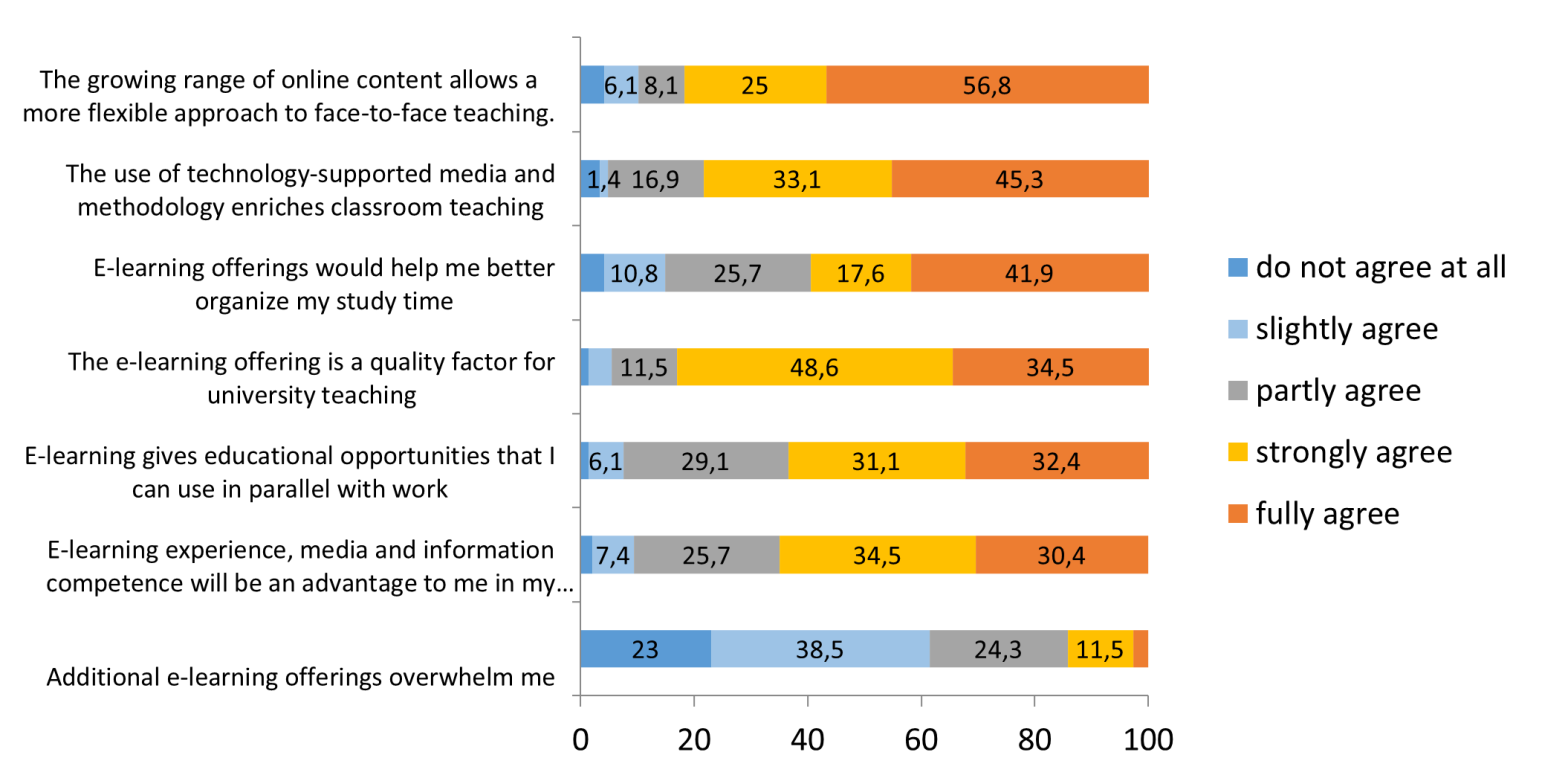
Assessments of the scope of e-learning for dental teaching (%)

When assessing the scope of e-learning factors for dental teaching, about 83 percent of the students agree that the e-learning offers are a quality factor for higher education institutions. Approximately 78 percent agree that using digital media and methodology enriches face-to-face teaching and about 82% state, that the growing range of online content allows a more flexible approach to face-to-face teaching. 64.9% agree, that e-learning experience etc. may be an advantage for the professional life and approximately the same amount of students agree to the option that e-learning enables educational opportunities parallel to work. Only a few students agree (14.2%) with the statement that e-learning offers overstrain them (see figure 4 (Fig. 4)). 

### 3.8. Problems and ideas

**Figure 5:**
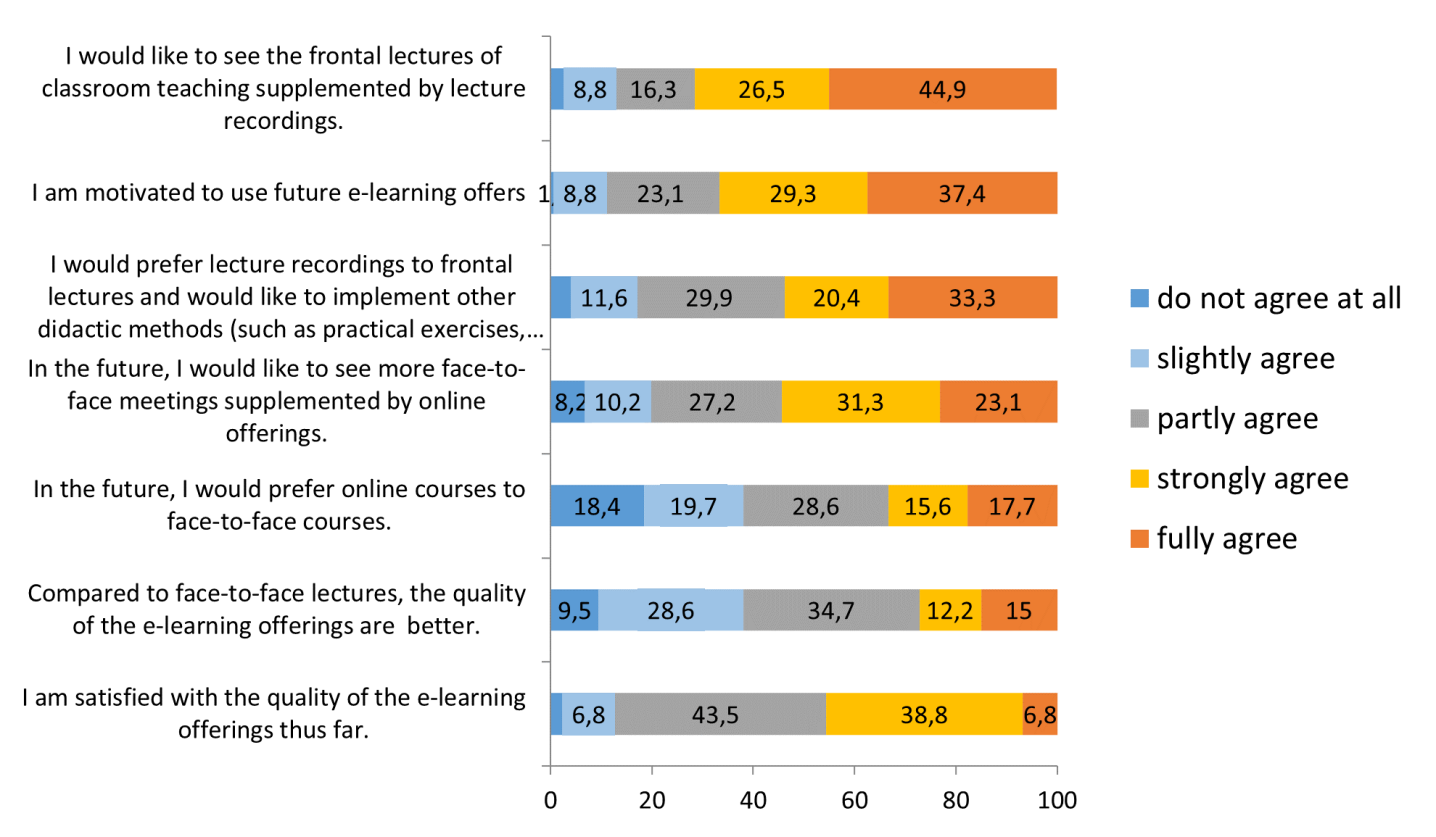
Students' wishes, problems and ideas about e-learning (%)

When concrete wishes and needs were mentioned, the offer to flank frontal lectures with digital media met with majority approval (71%). In this context, there was a demand to partially replace frontal lectures with recorded lectures to use the free attendance time for practical exercises (53.7%). About 67 percent of the students are motivated to use future e-learning offers, about 54 percent would strongly prefer more face-to face-meeting supplemented by online offerings and approximately 33 percent prefer online courses to face-to face courses. About 46 percent of the students are rather satisfied with the quality of the e-learning offerings and about 27 percent report, that the quality of e-learning offering is much better, than die quality of face-to face-lectures (see figure 5 (Fig. 5)).

## 4. Discussion

The aim of this study was to evaluate effective learning strategies from the student's perspective with a focus on different media. The results show that although students are proficient in the use of a variety of digital media, lectures and lecture recordings play a dominant role for all learning purposes. But depending on the learning purpose, students also favor different learning media. The learning of theoretical basics is supplemented by text- and e-books. For acquiring treatment-related theories and basics of dental treatment techniques, students additionally use videos and for the latter animations. Concerning learning more advanced practical skills, digital patient cases show high popularity. Finally students also supplement their preparations for exams with e-tests. Overall, no gender-related differences were found in the survey, while there were only minor semester-related differences.

The importance of e-tests is not surprising, as mock exams have a long tradition in medical education and replace learning with index cards. Lecture recordings seem to be essential to students as it was found for medical and dental students [13], [14]. One major reason for this could be that students can autonomously control and repeat the learning content by fast-forwarding and rewinding [15], [16]. For similar reasons, videos enjoy great popularity as tools for preparing for practical exams [17]. The possibility of visualizing facts is also particularly important here [16]. The more complex the content to be taught and the greater the prior knowledge in the subject area is, the greater the demand for digital media. Additionally, the more practice-oriented and complex the knowledge becomes, the more complex digital media, such as digital patient cases or simulations, appear to be reasonable [18]. At the same time, it was found that working with simulated patient cases could increase the self-confidence of dental students as a preparation for an intervention [19], [20].

Podcasts are currently considered a rather unconventional medium for acquiring knowledge in dentistry. However, they seem to be an interesting medium, already accepted for private use in terms of entertainment and news tracking, which could enrich the deepening of learning content [21]. The need for concise audio knowledge presentations, which can be received and internalized alongside other everyday activities, seems to be high. Previous studies clearly show the didactic added value of podcasts in medical teaching [21], [22]. Podcasts are suitable for both repeating subject content already heard and learning new concepts [23]. Podcasts are frequently used in the teaching of general medicine but less so in dental medicine. Future projects and teaching media developments could consider integrating this medium more strongly into didactic concepts.

Another important question in this study addresses the way students learn, their wishes and what consequences this has for the didactic concepts in teaching. Although dental studies are a formal learning program that is associated with high performance pressure to obtain a degree, the motivation to learn was found to be high. They have a positive attitude towards the use of digital media, especially when it comes to having more time available for practical exercises. They also see an advantage in the fact that through the use of digital media, lectures can be organized more flexibly and also the organization of their studies can be optimized. These results are confirmed by the existing literature [4], [8], [16].

For the conception of learning offers, these findings mean that attention should be given to keeping motivation high and offering appropriate incentives, e.g., by integrating gamification or reward systems in the digital learning offerings. According to Kerres, intrinsic motivation to learn is related to the need for competence and autonomy [24]. The learner wants to demonstrate competence and autonomy and experience self-efficacy [25], [26]. Accordingly, digital learning offerings should attach importance to meeting these needs (e.g., through exercises with feedback and interactions that enable self-direction in learning). Very important in this context is the principle of fit, according to which the task difficulty must be adapted to the competence level of the student so that he or she remains motivated. A favorable motivation develops when the student has the feeling that he or she can successfully master a task [26]. A careful formulation and presentation of concrete learning objectives is a basic prerequisite for satisfying this principle [27].

In addition, the survey shows that students like to use pictorial offers with a high degree of visualization and that a high number of illustrations/animations/graphics are desirable to support the learning process [28]. This result was also found in a current systematic review of digital undergraduate education in dentistry [15]. Here, it was shown that students prefer simulation training, especially for digital radiography and microscopy. In this context, future learning offerings should also implement corresponding impulses and influences from immersive learning. Additionally, studies show the didactic potential of this technology. Virtual reality training for preparing crowns or cavities was found to improve the students’ skills but still required instructions from tutors [15]. The embedding in a suitable didactic design is fundamental here. This technology can also play a decisive role with regard to virtual patient treatment [29], [30]. In this context, it must also be considered that within dental treatment, workflows are increasingly undergoing digitization. It is therefore important to learn how to operate various devices, such as an intraoral scanner or computer-aided design and manufacturing, at an early stage [15].

With regard to the limitations of this study, the limited number of participants should be mentioned. This may certainly be representative for the respective institution, however, it is questionable whether the results can be generalized or transferred to other disciplines. Furthermore, repeated reminders and motivations with regard to participation were dispensed due to the organizational effort involved.

In conclusion, there is a clear desire for a didactic restructuring and digital orientation of courses and concepts. A return to the status quo that prevailed before the coronavirus pandemic would certainly mean a major step backward. Instead, the wishes of the target group should be met, and suitable digitally flanked concepts should be created. The choice of media should also be considered, and attention should be given to which type of media should be used in which semester to convey which content.

## Acknowledgements

The authors would like to thank the members of the interclinical working group “Digitization”, which was funded by the Special Line Medicine - Funding Line Teaching by the Ministry of Science, Research and the Arts Baden-Württemberg, for recruiting students.

## Funding

This research received no external funding. The article processing charge was funded by the Baden-Wuerttemberg Ministry of Science, Research and Art and the University of Freiburg through the Open Access Publishing funding program.

## Notes

### Ethics statement

The study was approved by the Ethics Committee of the University of Freiburg on 13/10/2020 (EK No. 20-1159).

### Informed consent statement

Informed consent was obtained from all subjects involved in the study.

### Data availability statement

The data presented in this study are available on request from the corresponding author.

## Competing interests

The authors declare that they have no competing interests. 

## Supplementary Material

Questionnaire E-Learning V 1.0

## Figures and Tables

**Table 1:**
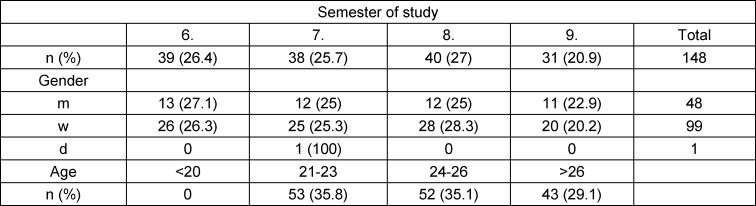
Demographics (semester, gender, age)

**Table 2:**
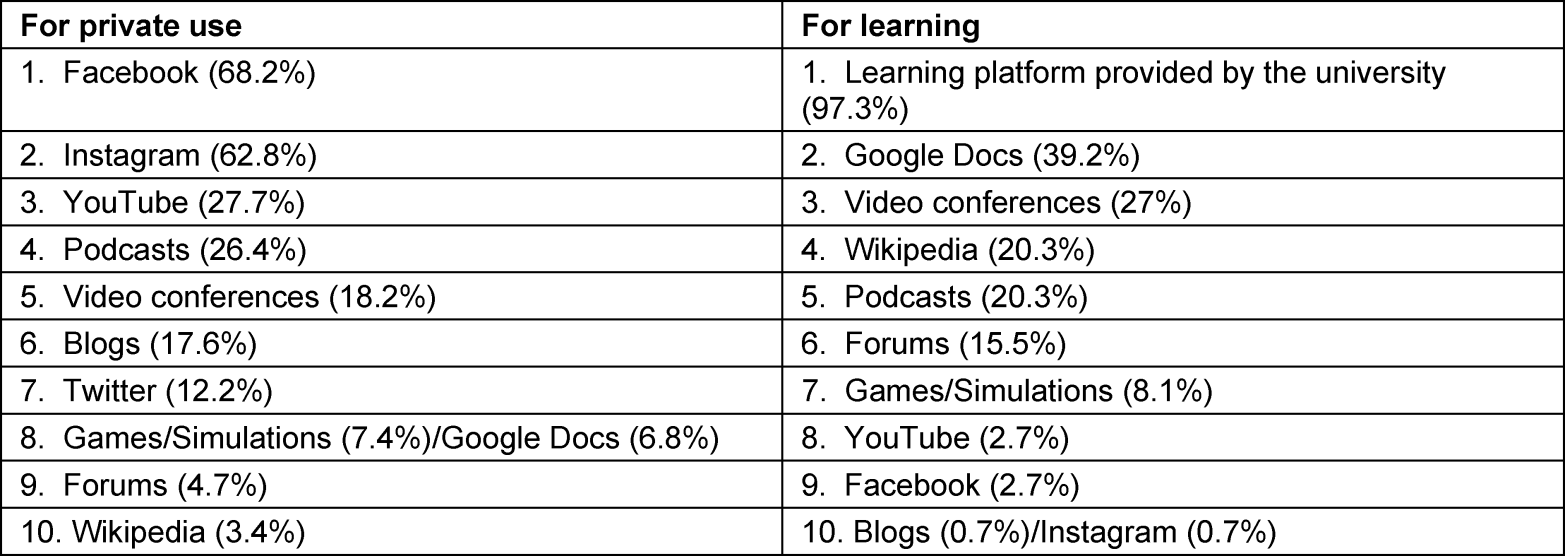
Top ten digital media for private and learning purposes (N=148)
