# Supplementary material for: Which digital learning strategies do undergraduate dentistry students favor? A questionnaire survey at a German university
Source: GMS J Med Educ. 2023 Jun 15;40(4):Doc49. doi: 10.3205/zma001631 (PMC10407589; doi:10.3205/zma001631)
Supplement: Questionnaire E-Learning V 1.0 [file JME-40-49-s-001.pdf]

Attachment 1: Questionnaire E-Learning V 1.0

1. General information

Date: ______________________

Gender:	male ☐	female ☐	diverse ☐						
Study semester:	1. ☐	2. ☐	3. ☐	4. ☐	5. ☐	6. ☐	7. ☐	8. ☐	9. ☐
Age (years):	< 20 ☐	21 – 23 ☐	24 – 26 ☐	> 26 ☐					

1. Questions on individual e-learning profile

	yes	no
**1.1 Have you already gained experience with e-learning offers (learning platforms, lecture recordings, teaching videos, etc.) outside of your studies (e.g. during your school time, training, private educational training, etc.)?**	☐	☐

If so, where and in what form? ______________________

	Daily	Weekly	Monthly	Occasionally (e.g. before exams)
**1.2 How often do you use the current e-learning offers in dental education?**	☐	☐	☐	☐

1.3 Which teaching media do you think are best for learning?

**Textbooks**	☐
**Lectures**	☐
**E-Learning offers**	☐

1.4 Which end devices are you most likely to use for learning?

**Tablet**	☐
**Smartphone**	☐
**Laptop/Desktop**	☐

1.5 Please choose which digital components you use and for what purpose.	Private	For learning purposes	Both	Not at all
E-Mail	☐	☐	☐	☐
Video conferencing (e.g. Skype)	☐	☐	☐	☐
Wikis (e.g. wikipedia)	☐	☐	☐	☐
Google Docs	☐	☐	☐	☐
Audio/Video Podcast	☐	☐	☐	☐
YouTube	☐	☐	☐	☐
Facebook	☐	☐	☐	☐
Twitter	☐	☐	☐	☐
Blogs	☐	☐	☐	☐
Learning platform (e.g. Ilias)	☐	☐	☐	☐
Educational games (Games, Simulationen)	☐	☐	☐	☐
Forums	☐	☐	☐	☐
Instagram	☐	☐	☐	☐

1.6 Which systems have you already worked with for learning purposes?	Yes	No		
Learning platform (e.g. Ilias)	☐	☐	If yes, which learning platform systems have you already used?	
Video conferencing, webinars (e.g. Adobe Connect, Zoom)	☐	☐	If yes, which of these services did you use for learning purposes?	
Wikis (e.g. Wikipedia)	☐	☐		
Systems for document creation and joint editing (e.g. Google Docs)	☐	☐		
Communication services (e.g. Skype, Whatsapp)	☐	☐	If yes, which communication services did you use for learning purposes?	
Social Media (e.g. Facebook, Twitter, Instagram)	☐	☐	If yes, which social media services did you use for learning purposes?	
Cloud services for document exchange (e.g. Dropbox)	☐	☐		
Databases for researching international scientific articles (e.g. Pubmed)	☐	☐		

**Digital services of Freiburg University Library (e.g. Katalog Plus)**	☐	☐		

1.7 The following statements describe different types of learning strate-gies. To what extent do they apply to the way you learn?

	Do not agree at all	Agree a little	Agree in part	Pretty much agree	Fully agree
**I like to study alone.**	☐	☐	☐	☐	☐
**I can motivate myself to learn.**	☐	☐	☐	☐	☐
**Digital teaching media support my learning process (e.g. videos)**	☐	☐	☐	☐	☐
**I think of concrete examples to which I can apply the material to be learned.**	☐	☐	☐	☐	☐
**I make diagrams and illustrations to structure the learning material.**	☐	☐	☐	☐	☐
**I try to visualise things.**	☐	☐	☐	☐	☐
**I find illustrations helpful for learning.**	☐	☐	☐	☐	☐

2. Assessing the scope of eLearning today and in the future

2.1 How do you assess the possibilities of e-learning for dental education?

	Do not agree at all	Agree a little	Agree in part	Pretty much agree	Fully agree
**The e-learning offer is a quality factor for university teaching.**	☐	☐	☐	☐	☐
**E-learning gives educational opportunities which I can use in parallel with work.**	☐	☐	☐	☐	☐
**E-learning experience, media and information competence will be an advantage to me in my (future) professional life.**	☐	☐	☐	☐	☐
**The use of technology-supported media and methodology enriches classroom teaching.**	☐	☐	☐	☐	☐
**The growing range of online content allows a more flexible approach to face-to-face teaching.**	☐	☐	☐	☐	☐
**E-learning offers would help me to organize my study time better.**	☐	☐	☐	☐	☐
**Additional e-learning offers overwhelm me.**	☐	☐	☐	☐	☐

3. Concrete wishes, problems and ideas

3.1 What are your concrete wishes. problems and ideas on this topic?

	Do not agree at all	Agree a little	Agree in part	Pretty much agree	Fully agree
**I am satisfied with the quality of the e-learning offers so far.**	☐	☐	☐	☐	☐
**In the future. I would like to see more face-to-face meetings supplemented by online offerings.**	☐	☐	☐	☐	☐
**In the future. I would prefer online courses to face-to-face courses.**	☐	☐	☐	☐	☐
**Compared to the face-to-face lectures. I find the quality of the e-learning offerings better.**	☐	☐	☐	☐	☐
**I would prefer lecture recordings to frontal lectures and would like to implement other didactic methods (practical exercises. group work. patient treatment) in the attendance time that is freed up.**	☐	☐	☐	☐	☐
**I would like to see the frontal lectures of classroom teaching supplemented by lecture recordings.**	☐	☐	☐	☐	☐
**I am motivated to use future e-learning offers**	☐	☐	☐	☐	☐

3.2 Which media type would you prefer to use for which specific learning purpose? (Multiple answers possible)

	Theoretical basic knowledge (e.g. anatomy. aetiology)	Treatment-related theory (e.g. material properties. patient communication)	Basics of dental treatment (ergonomics. professional tooth cleaning)	Further treatment (e.g. complex case planning. surgery. implantology)	Knowledge check (e.g. written exams, practical exams)
**Textbooks**	☐	☐	☐	☐	☐
**Lectures**	☐	☐	☐	☐	☐
**Lecture records**	☐	☐	☐	☐	☐
**E-Books**	☐	☐	☐	☐	☐
**Videos**	☐	☐	☐	☐	☐
**Digitale patient cases**	☐	☐	☐	☐	☐
**Animations/simulations**	☐	☐	☐	☐	☐
**Educational games**	☐	☐	☐	☐	☐
**Podcasts**	☐	☐	☐	☐	☐
**Online mock exams**	☐	☐	☐	☐	☐
**E-Portfolios**	☐	☐	☐	☐	☐
**Other interactive media (which ones?)**					
	☐	☐	☐	☐	☐
	☐	☐	☐	☐	☐

3.3 For which teaching contents would you wish for additional online offers?

**Exam preparation**	☐
**Course preparation**	☐
**Follow-up to the course**	☐
**Preparation for the practical exam**	☐
**Other**	☐

3.4 Please rate the following statements about the learning platform system Ilias.

	Do not agree at all	Agree a little	Agree in part	Pretty much agree	Fully agree
**The content of dental teaching is clearly presented on the learning platform and easy to find.**	☐	☐	☐	☐	☐
**The content on the learning platform system is complete.**	☐	☐	☐	☐	☐
**I would like to see the semester-based division of the teaching content maintained.**					
**With regard to navigation on the learning platform, I would like to have access to the respective teaching content starting from a patient case.**	☐	☐	☐	☐	☐
**I would like to see a topic-oriented listing of the contents independent of the subject element.**					

3.5 Please rate the following teaching strategies.

	Do not agree at all	Agree a little	Agree in part	Pretty much agree	Fully agree
**First, theoretical basics should be taught in the form of lectures and scripts. before these are deepened and applied to digitally prepared patient cases.**	☐	☐	☐	☐	☐
**I consider the teaching of theoretical content through e-learning offers (in addition to lectures/e-lectures) to be useful.**	☐	☐	☐	☐	☐

3.6 Please rate the following statements.

	Do not agree at all	Agree a little	Agree in part	Pretty much agree	Fully agree
**Based on digitally prepared patient cases. the individual basics are to be worked out in a topic-specific manner.**	☐	☐	☐	☐	☐

4. Personal Feedback

4.1 For which dental teaching content / topics would you like to see more media offers (e.g. videos showing surgical techniques)?

4.2 Please let us know any further wishes / ideas / suggestions.
